# Association between nutritional status, injury severity, and physiological responses in trauma patients

**DOI:** 10.3389/fphys.2024.1486160

**Published:** 2024-11-13

**Authors:** Linguo Niu, Yongning Zhang, Weihong Dai, Rixing Wang

**Affiliations:** ^1^ School of Emergency Trauma, Hainan Medical University, Haikou, China; ^2^ Department of Emergency, The Second Affiliated Hospital of Hainan Medical University, Haikou, China

**Keywords:** trauma severity, CONUT score, ISS, physiological abnormalities, predictive assessment, logistic regression, ROC analysis

## Abstract

**Purpose:**

To evaluate the predictive value of the Controlling Nutritional Status (CONUT) score and Injury Severity Score (ISS) in assessing physiological abnormalities and outcomes in trauma patients.

**Methods:**

A retrospective analysis was conducted on 354 trauma patients. Physiological parameters were assessed, including cardiovascular function, inflammatory response, liver and kidney function, and nutritional status. The CONUT score and ISS were calculated for each patient. Binary logistic regression was used to identify independent predictors of trauma severity. Receiver operating characteristic (ROC) curve analysis evaluated the predictive accuracy of the CONUT and ISS scores for adverse outcomes.

**Results:**

Severely injured patients exhibited more significant abnormalities in cardiovascular function, inflammatory response, liver and kidney function, and nutritional status compared to those with minor injuries. These patients had significantly higher CONUT scores. Logistic regression analysis identified white blood cell count, hemoglobin, and CONUT score as independent predictors of trauma severity. ROC analysis showed that both CONUT and ISS scores effectively predicted adverse outcomes, with ISS demonstrating better specificity.

**Conclusion:**

The CONUT and ISS scores are effective tools for predicting physiological abnormalities and adverse outcomes in trauma patients. Incorporating these scores into clinical practice may enhance prognostic assessments and improve management strategies for trauma patients.

## Highlights

• This study reports the initial application of the CONUT score and ISS score in the prognostic assessment of trauma patients.
• Our findings indicate that the CONUT score is an independent risk factor for severity in trauma patients.
• The study demonstrates that white blood cell counts can predict the mortality risk in trauma patients.
• This study finds that age and ISS score are significant predictors of mortality in trauma patients.
• This research provides new theoretical foundations and tools for risk assessment and clinical decision-making in trauma patients.

## Introduction

In recent years, trauma has become a significant public health issue threatening people’s health (Worley, 2019). In our country, trauma-related deaths account for 10% of total mortality annually and have become the leading cause of death among people under 45 years old (Moore et al., 2021; Harper et al., 2024). Regarding the three peaks of post-traumatic deaths, they are divided into immediate, early, and late deaths (Roberts et al., 2021; Lang et al., 2023). Within hours of injury, nearly 80% of trauma deaths are immediate or early, often caused by severe brain injuries or significant blood loss (Du et al., 2023; Nozari et al., 2023). Effective hemostasis and resuscitation can prevent early deaths in trauma patients (Moore et al., 2021; Kuo and Palmer, 2022; Eissa et al., 2022). Late deaths occur days or weeks after the initial injury, with severe infections and multi-organ failure being the main causes of late deaths in trauma patients. Therefore, predictive assessment for trauma patients is crucial for improving treatment outcomes, optimizing resource allocation, and reducing mortality rates. However, the complexity and variability of trauma patients’ conditions present numerous challenges in predictive assessment. Therefore, accurate prognostic evaluation of trauma patients is crucial for improving treatment outcomes, optimizing resource allocation, and reducing mortality. Although various trauma scoring systems have been developed, a gap remains in integrating nutritional status with injury severity to predict outcomes, which is essential for a more comprehensive trauma prognosis. However, the complexity and variability of trauma patients’ conditions present significant challenges for prognosis evaluation. Therefore, identifying and applying multifaceted prognostic tools is particularly important.

The severity of injuries in trauma patients is a critical factor affecting their prognosis. Currently, the abbreviated injury scale (AIS) and the injury severity score (ISS) are the recommended trauma scoring methods within hospitals (Herren et al., 2023; Marchant et al., 2024). The AIS score is based on anatomical features, dividing the human body into six regions to assess the severity of injuries in trauma patients (Kang et al., 2021; Lee et al., 2022). The ISS score is calculated by summing the squares of the highest AIS values for the three most severely injured body regions (Birri et al., 2022; Mora-Boga et al., 2022; Yamamoto et al., 2023). However, these scoring systems have limitations; for example, the AIS and ISS scores are primarily based on anatomical injuries and may not fully reflect the physiological condition and prognosis of the patients. Nutritional status is closely related to the clinical prognosis of trauma patients, and malnutrition can exacerbate the pathophysiological response following trauma, affecting treatment outcomes and prognosis. Therefore, using a combination of various scoring systems for assessment might be more accurate and reliable.

In 2005, Professor Ignacio and others proposed the controlling nutritional status (CONUT) score, which includes three biomarkers: serum albumin, absolute peripheral lymphocyte count, and total cholesterol concentration. The CONUT score is a simple, efficient, and objective nutritional assessment tool that can detect and monitor the nutritional and immune status of hospitalized patients early on, serving as an effective standard for predicting patient outcomes (Di Vincenzo et al., 2023; Li et al., 2022; Ishii et al., 2021). In malnutrition, the nutritional support for the body’s organ systems is insufficient, preventing the body from performing its basic physiological functions. It can exacerbate oxidative stress damage, inflammatory responses, and catabolic metabolism within the body, affecting treatment outcomes and leading to prolonged hospital stays and increased mortality rates (Gu et al., 2023; Ni et al., 2023; Li et al., 2023). However, the application of the CONUT score in trauma prognosis evaluation has not been fully explored, particularly in combination with the existing ISS score. This study aims to bridge this gap by assessing the predictive value of integrating these two scoring systems.

Currently, clinical research on the CONUT score primarily focuses on cardiovascular diseases, malignant tumors, cranial injuries, sepsis, gastrointestinal surgeries, orthopedic postoperative prognoses, and complications. However, studies on applying the CONUT score and ISS score in the prognostic assessment of trauma patients are limited and often involve small samples, leading to some controversy in the results. Some studies indicate that the CONUT score and ISS score significantly predict adverse outcomes in trauma patients, but others have not confirmed their statistical significance. Moreover, existing research often concentrates on assessing a single scoring system and lacks a systematic study combining both scores. Therefore, it is necessary to conduct large-scale, multi-center studies to further verify the application value of the CONUT score and ISS score in trauma prognostic assessments and to explore their relationship with the severity of trauma and the risk of death using relevant physiological indicators.

The study validated the effectiveness of combining the CONUT and ISS scores in diagnosing trauma patients. These two scoring tools can serve as independent predictors of adverse outcomes in trauma patients, enabling clinicians to assess patient survival prognosis more comprehensively. This combination not only provides strong support for risk stratification and treatment decision-making but also offers a basis for personalized and precise management of trauma patients. Specifically, the CONUT score effectively reflects the patient’s nutritional status and immune function, while the ISS score accurately assesses the severity of anatomical injuries. Integrating these tools offers clinicians a more comprehensive predictive evaluation method, especially in emergency medical settings, helping optimize resource allocation, improve patient survival rates, and enhance quality of life.

The findings of this study provide new tools for clinical trauma practice. They aid in the early identification of high-risk patients and improve patient prognosis through nutritional intervention and personalized treatment. These findings hold significant clinical value, especially for physicians in resource-limited healthcare settings, as they can rely on these simple yet effective scoring tools to optimize treatment strategies and significantly enhance trauma patient outcomes.

## Materials and methods

### Patient inclusion and exclusion criteria

**FIGURE 1:**
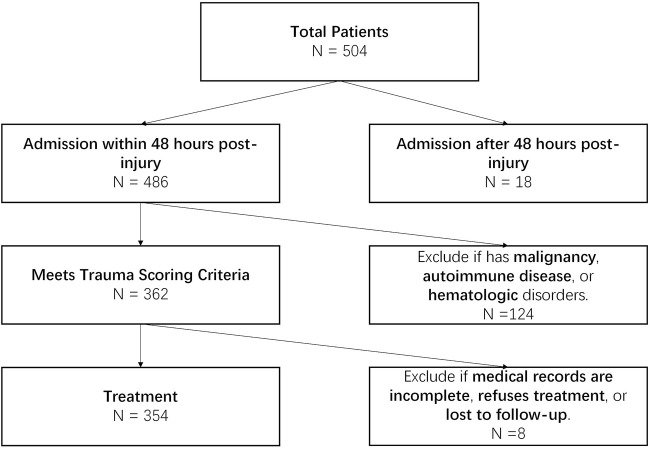
Patient inclusion and exclusion process diagram.

This study is a retrospective analysis that collected clinical data from 354 trauma patients admitted to a tertiary hospital in this city between January 2021 and June 2023. The study aimed to minimize potential bias by including patients with diverse demographic backgrounds and varying degrees of injury severity. Particular attention was given to covering different age groups and genders, as these factors may introduce confounding effects on trauma outcomes. Inclusion criteria: (1) age and gender not restricted; (2) admitted within 48 h after injury; (3) clear injury status and ISS score conducted. Patients meeting the trauma score criteria were included. All patients were screened to ensure the absence of severe comorbidities that could affect trauma outcomes, such as malignant tumors or immune system disorders. Exclusion criteria: (1) concomitant malignancies, autoimmune diseases, blood disorders, etc.,; (2) incomplete medical records, refusal of any treatment, or loss to follow-up (Figure 1). These exclusion criteria aimed to minimize the interference of comorbidities with study outcomes and ensure data integrity, guaranteeing statistical analysis’s accuracy and reliability.

### Research methods

Clinical indicators of patients: The collected information includes the gender, age, mean arterial pressure (MAP), comorbidities, site of injury, patient’s prognosis, and other laboratory indicators of hospitalized patients, including highly sensitive C-reactive protein (CRP), white blood cells, hemoglobin, platelets, neutrophil granulocyte (NEUT%), absolute lymphocyte count, monocyte (Mon%), total bilirubin (TBil), blood urea nitrogen, blood creatinine, serum albumin, serum total cholesterol, aspartate aminotransferase (AST), alanine aminotransferase (ALT), low-density lipoprotein (LDL), and high-density lipoprotein (HDL), etc. The patient’s ISS and CONUT scores are calculated based on the vital signs, injury site, and laboratory tests recorded within 24 h of admission. Patients are categorized into different groups based on the ISS score, with ≤16 points classified as the minor injury group and >16 points as the severe injury group. Patients are divided into survival and death groups based on their 28-day survival status.

CONUT score: Collect serum albumin, absolute lymphocyte count in peripheral blood, and serum total cholesterol concentration of the selected patients. Based on the calculation of the above three parameters, the specific criteria and assessment method are as follows, as detailed in Supplementary Table S1. The three parameters represent protein reserves, immune defense, and energy consumption, which can accurately reflect the body’s nutritional status and immune function. A higher score indicates a poorer nutritional status of the patient.

ISS score: The human body is divided into 6 regions based on anatomical features: head and neck, face, chest, abdomen, limbs, and body surface. A score of 1 to 6 is assigned based on the extent of injury in the respective regions, representing minor, moderate, serious, severe, critical, and the most severe injuries (currently untreatable). The square of the highest AIS value of the three most severely injured body parts is summed to calculate the ISS score (Supplementary Table S2). The ISS score ranges from 1 to 75, where an ISS score >16 and ≤25 is defined as a more severe injury with a less than 10% chance of death; an ISS score >25 and <50 is defined as a severe injury with a certain risk of death; an ISS score ≥50 is defined as a critical injury with a very low survival probability, almost on the brink of death.

### Statistical methods

SPSS 26.0 software was used for data processing and analysis. Frequency data were expressed as rates (%), and the chi-square test was used. Normally distributed continuous data were expressed as mean ± standard deviation (Mean ± SD) and analyzed using the t-test or corrected t-test. Non-normally distributed continuous data were expressed as median (quartiles) [M (P25, P75)] and analyzed using the Mann-Whitney U test. Univariate and multivariate logistic regression analyses were conducted to control for potential confounding variables. In the multivariate analysis, age, gender, and comorbidities were included to eliminate their potential impact on patient outcomes. The study assumed a linear relationship between variables and the data followed a normal distribution. For variables that did not conform to a normal distribution, non-parametric testing methods were applied to ensure the robustness of the analysis. The multivariate regression analysis assumed that the residuals were independently and identically distributed, with no multicollinearity present.

Initially, univariate analysis was performed to screen out variables related to prognosis. For variables that showed statistical significance in univariate analysis, further multivariate logistic regression analysis was conducted to control potential confounding factors and determine independent risk factors. The selection of variables was based on statistical significance and clinical relevance to ensure the robustness of the model. In the regression analysis, the inclusion criterion was set at *p* < 0.05, and odds ratios (OR) with 95% confidence intervals (CI) were calculated to assess the strength of their association with prognosis. By incorporating confounding factors into the regression model, this study aimed to isolate the effect of key independent variables on patient outcomes, thereby enhancing the reliability of the analysis results.

The CONUT score’s predictive ability for adverse outcomes in trauma patients was evaluated using the receiver operating characteristic (ROC) curve, with the area under the curve (AUC) used to quantify the model’s discriminative capacity. The optimal threshold of the ROC curve was determined using the Youden Index, calculated as the maximum value of sensitivity plus specificity minus 1. This threshold represents the point that achieves the best balance between sensitivity and specificity.

The cutoff points were determined based on statistical analysis results and clinical relevance. The optimal cutoff values for the CONUT and ISS scores were selected according to the statistical findings and by considering existing literature and practical clinical applications. For example, the CONUT score cutoffs were set at 2, 4, and 8, reflecting different degrees of malnutrition. The ISS score cutoffs were chosen at 16, 25, and 50, corresponding to mild, moderate, severe, and critical trauma levels. These thresholds effectively stratify patients by risk level and assist in formulating personalized treatment plans. In all statistical tests, a p-value <0.05 was considered statistically significant.

## Results

### Baseline clinical characteristics for evaluating the effectiveness of CONUT and ISS scores in trauma patients

**FIGURE 2:**
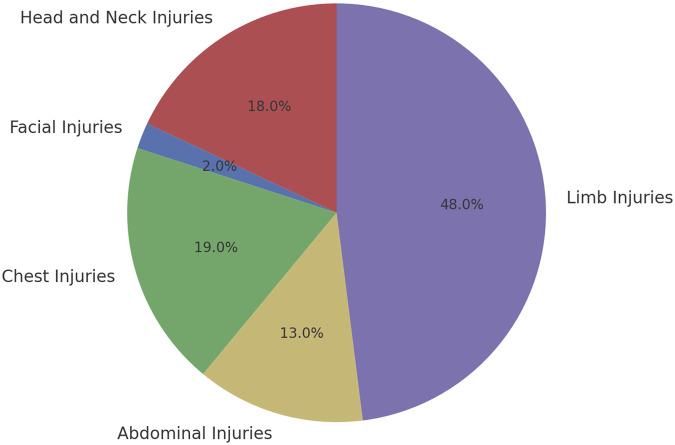
Distribution of single injuries in trauma patients.

**FIGURE 3:**
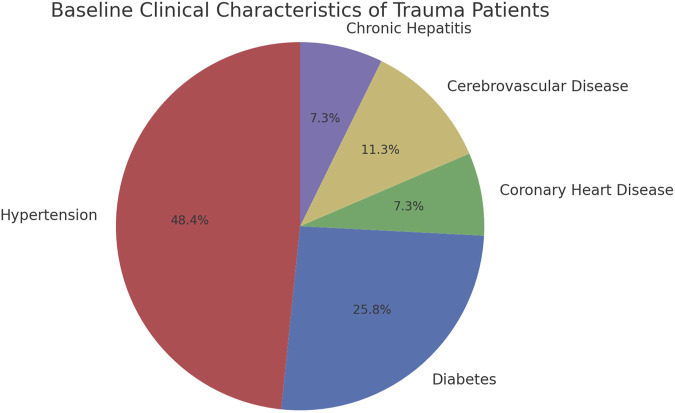
Baseline clinical characteristics of trauma patients evaluating the effectiveness of Conut and ISS scores.

Among the 354 patients included in this study, there were 208 cases of single-site injuries and 146 cases of multiple injuries. In the single-site injuries, there were 37 cases of head and neck injuries, 4 cases of facial injuries, 39 cases of chest injuries, 28 cases of abdominal injuries, and 100 cases of limb injuries, as well as 146 cases of multiple injuries. There were 316 patients discharged with improvement and 38 deaths (Figure 2; Supplementary Table S3). The average age of the patients was 50.7 ± 17.3 years, with 268 males (75.7%) and 86 females (24.3%). Among them, 47 patients (13.3%) had hypertension, 25 patients (7.1%) had diabetes, 7 patients (2.0%) had coronary heart disease, and 7 patients (2.0%) had chronic hepatitis (Figure 3); 11 patients (3.1%) had a history of cerebrovascular diseases, and the median length of hospital stay was 13 days (Supplementary Table S4).

### Comparison of clinical indicators among patients classified by the severity of injury

This study divided patients into the mild injury group (n = 196) and the severe injury group (n = 158). By comparing the baseline characteristics of the two groups of patients, it was found that the heart rate in the mild injury group was significantly lower than that in the severe injury group (82 beats/min vs. 89 beats/min, *p* < 0.001), while the MAP was significantly higher than that in the severe injury group (97.64 ± 11.97 mmHg vs. 91.23 ± 22.79 mmHg, *p* = 0.001). Additionally, the white blood cell count, NEUT%, AST, ALT, blood urea nitrogen, and creatinine levels in the mild injury group were significantly lower than those in the severe injury group (*p* < 0.05), indicating that patients with severe injuries experienced more severe inflammation and liver and kidney function damage. Although the level of highly sensitive CRP was higher in the severe injury group, the difference between the two groups was not statistically significant (*p* > 0.05) (Table 1).

**TABLE 1:** Baseline characteristics of patients in the mild injury group and severe injury.

Indicator	Mild injury group (*n* = 196)	Severe injury group (*n* = 158)	*t*/*χ* ^*2*^	*p*
Gender			−1.340	0.180
Male	143 (73.0)	125 (79.1)		
Female	5 (27.0)	33 (20.9)		
Age (years)	51.56 ± 18.24	49.63 ± 16.21	1.038	0.300
Heart rate (beats per minute)	82 (78,90)	89 (78,108)	−4.019	<0.001
MAP (mmHg)	97.64 ± 11.97	91.23 ± 22.79	3.375	0.001
CRP (mg/L)	5.45 (1.89,16.73)	8.22 (1.61,24.82)	−1.328	0.184
White blood cells (×10^9^/L)	9.86(8.06,12.41)	13.01 (9.32,17.35)	−5.355	<0.001
White blood cells (g/L)	132 (118,144)	119 (96,138)	−4.812	<0.001
Platelets (×10^9^/L)	214 (179,251)	188 (142,246)	−3.374	0.001
NEUT% (%)	77.10 (70.53,84.70)	86.05 (80.88,89.70)	−7.265	<0.001
Mon% (%)	6.25 (5.10,8.00)	5.50 (4.40,6.80)	−3.982	<0.001
TBil(μmol/L)	14.6(10.6,18.8)	14.2 (9.2,19.4)	−0.112	0.911
AST(U/L)	28 (20,38)	53 (33,115)	−8.607	<0.001
ALT(U/L)	24 (15,36)	33 (20,73)	−4.669	<0.001
Blood urea nitrogen (μmol/L)	4.9 (3.8,6.4)	5.6 (4.5,7.1)	−3.786	<0.001
Creatinine (mmol/L)	68 (56,79)	70 (57,92)	−2.128	0.033
LDL (mmol/L)	2.57 (2.00,3.16)	2.05 (1.36,2.82)	−5.389	<0.001
HDL (mmol/L)	1.28 (1.10,1.54)	1.07 (0.82,1.40)	−4.718	<0.001
CONUT score	4 (3,5)	6 (5,8)	−7.821	<0.001

Note: CONUT, controlled nutritional status assessment; ISS, injury severity score; CRP, C-reactive protein; MAP, mean arterial pressure; NEUT%, neutrophil percentage; Mon%, Monocyte percentage; TBil, Total bilirubin; AST, aspartate aminotransferase; ALT, alanine aminotransferase; LDL, Low-density lipoprotein; HDL, High-density lipoprotein.

Regarding nutritional status and blood lipid metabolism, the levels of LDL and HDL were significantly higher in the mild injury group than in the severe injury group (*p* < 0.001). Additionally, the CONUT score was significantly lower in the mild injury group than in the severe injury group (4 vs. 6, *p* < 0.001), indicating more severe malnutrition and lipid metabolism disorders in patients with severe injuries. Furthermore, the levels of hemoglobin, platelets, and Mon% were significantly higher in the mild injury group compared to the severe injury group (*p* < 0.05) (Table 1). It is worth noting that there were no significant differences in gender, age, and TBil levels between the mild injury group and the severe injury group (*p* > 0.05), suggesting that these factors are not significantly correlated with the severity of injuries.

In conclusion, patients with severe injuries exhibited more significant abnormalities in the cardiovascular system, inflammatory response, liver and kidney function, blood lipids, and nutritional status, while gender and age had a minor impact on the severity of injuries.

### White blood cells, hemoglobin, and CONUT score are independent risk factors for the severity of trauma patients

**FIGURE 4:**
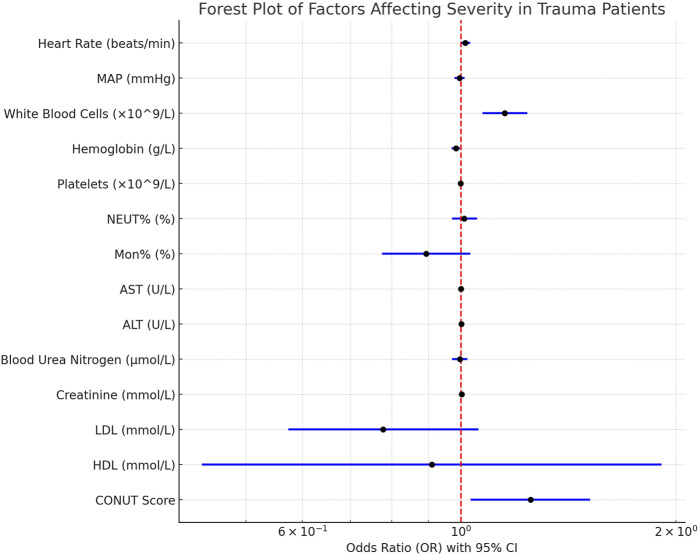
Forest plot of predictors affecting severity in trauma patients.

We also conducted binary logistic regression analysis on trauma patients’ heart rate, MAP, white blood cell count, hemoglobin, platelet count, NEUT%, Mon%, AST, ALT, blood urea nitrogen, creatinine, LDL, HDL, and CONUT score to evaluate the impact of these factors on the severity of trauma patients. The results indicated that white blood cell count, hemoglobin, and CONUT score were independent risk factors for the severity of trauma patients (*p* < 0.05). Specifically, white blood cell count (β = 0.141, OR = 1.152, 95% CI: 1.072–1.238, *p* < 0.001) and CONUT score (β = 0.224, OR = 1.251, 95% CI: 1.032–1.516, *p* = 0.022) were positively correlated with the severity of trauma patients, meaning that higher white blood cell count and CONUT score were associated with greater severity of trauma. On the other hand, hemoglobin (β = −0.017, OR = 0.984, 95% CI: 0.970–0.997, *p* = 0.019) showed a negative correlation with the severity of trauma patients, meaning that higher hemoglobin levels were associated with lower severity of trauma (Figure 4). Other indicators such as heart rate, MAP, platelet count, NEUT%, Mon%, AST, ALT, blood urea nitrogen, creatinine, LDL, and HDL were included in the analysis but did not exhibit significant independent correlations (*p* > 0.05) (Table 2). In conclusion, white blood cell count, hemoglobin, and CONUT score are important in assessing the severity of trauma patients.

**TABLE 2:** Binary logistic regression analysis of factors influencing the severity of trauma patients.

Indicator	*p* value	*OR* value	95%CI
Heart rate (beats per minute)	0.090	1.014	0.998 ∼ 1.031
MAP (mmHg)	0.579	0.995	0.979 ∼ 1.012
White blood cells (×10^9^/L)	<0.001	1.152	1.072 ∼ 1.238
Hemoglobin (g/L)	0.019	0.984	0.970 ∼ 0.997
Platelets (×10^9^/L)	0.577	0.999	0.995 ∼ 1.003
NEUT% (%)	0.590	1.011	0.971 ∼ 1.054
Mon% (%)	0.125	0.894	0.775 ∼ 1.031
AST (U/L)	0.893	1.000	0.997 ∼ 1.002
ALT (U/L)	0.587	1.001	0.997 ∼ 1.006
Blood urea nitrogen (μmol/L)	0.762	0.996	0.972 ∼ 1.021
Creatinine (mmol/L)	0.651	1.002	0.994 ∼ 1.010
LDL (mmol/L)	0.110	0.778	0.573 ∼ 1.058
HDL (mmol/L)	0.805	0.911	0.434 ∼ 1.910
CONUT score	0.022	1.251	1.032 ∼ 1.516

### White blood cells, hemoglobin, and CONUT score are important factors that Influence the prognosis of trauma patients

**FIGURE 5:**
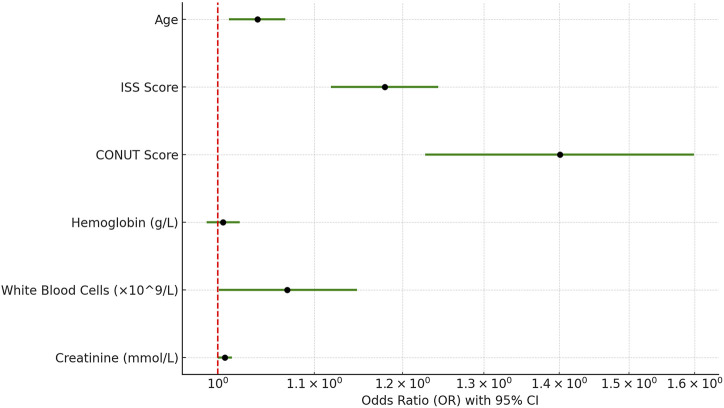
Forest plot of predictors affecting prognosis in trauma patients.

Among the 354 trauma patients included in this study, 316 patients (89.3%) survived, while 38 patients (10.7%) succumbed. Through univariate analysis of these patients, several key clinical indicators were found to significantly impact prognosis. The results showed that the mortality rate was 1.7% in patients with low CONUT scores and 9.0% in patients with high CONUT scores. There were no significant differences between the survival and deceased groups of patients in terms of gender, CRP, Mon%, TBil, ALT, LDL, history of hypertension, history of coronary heart disease, history of diabetes, history of chronic hepatitis, and history of cerebrovascular diseases (*p* > 0.05). It indicates that these factors have a minor impact on the prognosis of trauma patients. Compared to the deceased group, patients in the survival group had significantly lower age, ISS score, CONUT score, heart rate, white blood cells, NEUT%, AST, blood urea nitrogen, and creatinine, while MAP, hemoglobin, platelets, HDL, and length of hospital stay were significantly higher in the survival group. These differences were statistically significant (*p* < 0.05). It suggests that these clinical indicators have significant reference value in predictive assessment. Specifically, patients in the survival group had a lower average age (49.8 ± 17.1 years vs. 57.5 ± 18.0 years, *p* = 0.010), lower ISS score (13 vs. 29, *p* < 0.001), lower CONUT score (5 vs. 7, *p* < 0.001), lower heart rate (85 beats/min vs. 97 beats/min, *p* = 0.033), lower white blood cell count (10.51 vs. 13.77, *p* < 0.001), lower NEUT% (82% vs. 86%, *p* = 0.003), lower AST level (32 vs. 51, *p* = 0.001), lower blood urea nitrogen level (5.14 vs. 5.74, *p* = 0.002), and lower creatinine level (67 vs. 93, *p* < 0.001). Additionally, they had higher MAP (95.7 ± 15.7 mmHg vs. 87.4 ± 29.9 mmHg, *p* = 0.008), higher hemoglobin level (129 vs. 116, *p* = 0.017), higher platelet count (210 vs. 173, *p* = 0.005), higher HDL level (1.21 vs. 1.01, *p* = 0.015), and a longer length of hospital stay (14 days vs. 5 days, *p* < 0.001) (Figure 5; Table 3). In conclusion, white blood cell count, hemoglobin, and CONUT score are important factors that influence the prognosis of trauma patients.

**TABLE 3:** Univariate analysis of factors influencing the prognosis of trauma.

Grouping	Survival group (*n* = 316)	Death group (*n* = 38)	*t/χ* ^*2*^	*p*
Gender			0.355	0.551
Male	237 (75.0%)	31 (81.6%)		
Female	79 (25.0%)	7 (18.4%)		
Age (years)	49.8 ± 17.1	57.5 ± 18.0	−2.584	0.010
ISS score	13 (9,20)	29 (22,34)	−7.615	<0.001
CONUT score	5 (3,6)	7 (5,9)	−4.932	<0.001
Heart rate (beats per minute)	85 (78,96)	97 (75,116)	−2.135	0.033
MAP(mmHg)	95.7 ± 15.7	87.4 ± 29.9	2.679	0.008
CRP(mg/L)	7.66 (1.96,21.13)	3.60 (1.25,13.86)	−1.859	0.063
White blood cells (×10^9^/L)	10.51(8.12,13.92)	13.77 (11.79,18.93)	−3.856	<0.001
Hemoglobin (g/L)	129 (110,141)	116 (90,139)	−2.379	0.017
Platelets (×10^9^/L)	210 (166,250)	173 (124,278)	−2.817	0.005
NEUT% (%)	82 (74,87)	86(81,90)	−2.921	0.003
Mon% (%)	6.0 (4.8,7.5)	5.4 (4.0,7.0)	−1.644	0.100
TBil (μmol/L)	14.6 (10.5,19.5)	14.2 (7.9,18.5)	−1.075	0.283
AST (U/L)	32 (24,63)	51 (34,84)	−3.181	0.001
ALT(U/L)	26 (17,45)	24 (17,50)	−0.214	0.831
Blood urea nitrogen (μmol/L)	5.14 (4.00,6.66)	5.74 (5.08,9.09)	−3.119	0.002
Creatinine (mmol/L)	67 (56,81)	93 (67,130)	−4.547	<0.001
LDL (mmol/L)	2.43 (1.78,3.07)	2.11(1.45,2.85)	−1.763	0.078
HDL (mmol/L)	1.21 (0.97,1.49)	1.01 (0.74,1.37)	−2.442	0.015
Hypertension			0.978	0.323
yes	40 (12.7%)	7 (18.4%)		
no	276 (87.3%)	31 (81.6%)		
Diabetes			2.410	0.121
yes	20 (6.3%)	5 (13.2%)		
no	296 (93.7%)	33 (86.8%)		
Coronary heart disease			0.094	0.759
yes	6 (1.9%)	1 (2.6%)		
no	310 (98.1%)	37 (97.4%)		
Cerebrovascular disease			0.032	0.858
yes	10 (3.2%)	1 (2.6%)		
no	306 (96.8%)	37 (97.4%)		
Chronic hepatitis			0.859	0.354
yes	7 (2.2)	0 (0.0)		
no	309 (97.8)	38 (100.0)		
Length of hospital stay (days)	14 (8,23)	5 (1,25)	−3.347	<0.001

### Age, CONUT score, ISS score, white blood cells, and creatinine are independent risk factors for 28-day mortality in trauma patients

In this study, by including the variables with statistical significance from univariate analysis into a binary logistic regression model to eliminate the effects of confounding factors, the analysis results revealed that age, CONUT score, ISS score, white blood cells, and creatinine were independent risk factors for 28-day mortality in trauma patients (*p* < 0.05). Specifically, increasing age significantly raised the risk of 28-day mortality in trauma patients (β = 0.039, OR = 1.040, 95% CI: 1.011–1.069, *p* = 0.007), indicating that higher age is associated with a higher risk of mortality. Moreover, higher ISS scores (β = 0.164, OR = 1.179, 95% CI: 1.118–1.243, *p* < 0.001) and CONUT scores (β = 0.337, OR = 1.401, 95% CI: 1.227–1.599, *p* < 0.001) significantly increased the risk of death, suggesting that severity of injury and malnutrition are important factors influencing the prognosis. In addition, an increase in white blood cell count (β = 0.069, OR = 1.071, 95% CI: 1.001–1.147, *p* = 0.047) reflects the close relationship between the intensity of the inflammatory response and patient prognosis, while a higher creatinine level (β = 0.007, OR = 1.007, 95% CI: 1.000–1.014, *p* = 0.035) indicates the significant impact of kidney function damage on patient prognosis (Table 4). These results indicate that age, CONUT score, ISS score, white blood cells, and creatinine are independent risk factors for 28-day mortality in trauma patients.

**TABLE 4:** Binary logistic regression analysis of factors influencing the prognosis of trauma patients.

Indicator	*p* value	*OR* value	95%CI
Age	0.007	1.040	1.011 ∼ 1.069
ISS score	<0.001	1.179	1.118∼1.243
CONUT score	<0.001	1.401	1.227 ∼ 1.599
Hemoglobin (g/L)	0.541	1.005	0.989 ∼ 1.022
White blood cells (×10^9^/L)	0.047	1.071	1.001 ∼ 1.147
Creatinine (mmol/L)	0.035	1.007	1.000 ∼ 1.014

### The predictive value of CONUT score and ISS score for adverse outcomes in trauma patients

**FIGURE 6:**
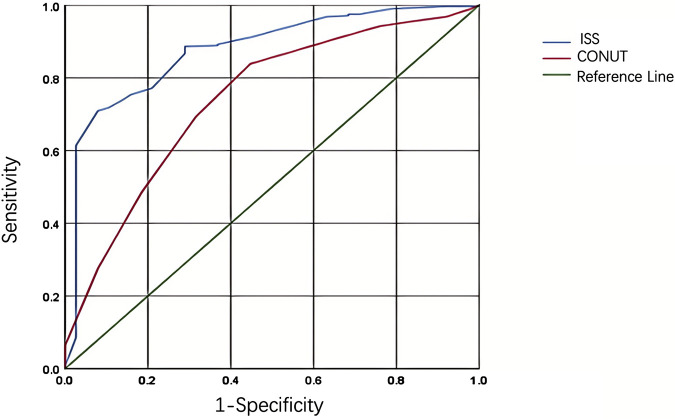
Roc curves analysis of adverse prognosis in trauma patients based on CONUT score and ISS score.

In this study, the predictive value of the CONUT score and ISS score for adverse outcomes in trauma patients was evaluated through ROC curve analysis. The results showed that the CONUT and ISS scores have relatively high predictive capabilities. As seen from Table 5 and Figure 6, the AUC of the CONUT score was 0.741 (95% CI: 0.657∼0.826) with a cut-off value of 5.5. At this cut-off value, the sensitivity of the CONUT score was 69.3%, and the specificity was 68.4%. It indicates that when the CONUT score exceeds 5.5, there is a higher likelihood of adverse outcomes for the patients, and this score demonstrates a good balance in predicting adverse outcomes. The AUC of the ISS score was 0.876 (95% CI: 0.819 ∼ 0.934), with a cut-off value of 17.5. At this cut-off value, the sensitivity of the ISS score was 70.9%, and the specificity was 92.1%. It suggests that the ISS score has higher specificity in predicting adverse outcomes, implying that when the ISS score exceeds 17.5, there is a significant increase in the likelihood of adverse outcomes for the patients.

**TABLE 5:** Predictive value of CONUT score and ISS score for adverse outcomes in trauma.

Indicator	AUC	*p*	95%CI	Sensitivity	Specificity
CONUT score	0.741	<0.001	0.657∼0.826	69.3	68.4
ISS score	0.876	<0.001	0.819∼0.934	70.9	92.1

In conclusion, the CONUT and ISS scores have high predictive value for adverse outcomes in trauma patients, with the ISS score demonstrating higher specificity. These findings are of significant reference value for clinical doctors in assessing the prognosis of trauma patients.

## Discussion

Trauma is one of the leading causes of death and disability worldwide, with high incidence and mortality rates in many countries (Griswold et al., 2022; GBD 2021 Diseases and Injuries Collaborators, 2024; McCrea et al., 2021). In our country, trauma-related deaths account for 10% of all deaths annually and have become the primary cause of death among individuals under 45 years old (Dixon et al., 2024; Im et al., 2023). There are three peaks in post-traumatic mortality: immediate, early, and late deaths. In the hours following injury, nearly 80% of trauma deaths are either immediate or early, typically due to severe brain injury or significant blood loss. Effective hemostasis and resuscitation measures can prevent early deaths (Moore et al., 2021; Sperry et al., 2023). Late deaths occur days or weeks after the initial injury, primarily due to severe infections and multi-organ failure (Marsden and Tuma, 2023; Starr et al., 2024; Battaglini et al., 2021). Additionally, the increase in traffic and industrial accidents, driven by modern transportation and industrial developments, has led to a higher frequency of traumatic incidents. Given this grave situation, accurate prognostic assessment tools are crucial for optimizing treatment plans and improving patient survival rates. However, the conditions of trauma patients are complex and variable, and existing prognostic assessment tools have their limitations. Therefore, this study, through analyzing data from 354 trauma patients, explores the effectiveness of the CONUT score and ISS score in trauma prognostic assessments, guiding clinical treatment.

This study found that the CONUT and ISS scores can effectively predict adverse outcomes in trauma patients. Compared to previous research, our results further validate the effectiveness of these two scoring systems. For example, some studies have shown that the ISS score can reflect the severity of trauma well, but its accuracy may be limited under specific circumstances. The CONUT score, traditionally used as a nutritional assessment tool primarily in patients with cardiovascular diseases and malignancies, has been less frequently applied to trauma patients. Our findings demonstrate that the CONUT score can also effectively predict the prognosis of trauma patients, thereby broadening its application scope.

**FIGURE 7:**
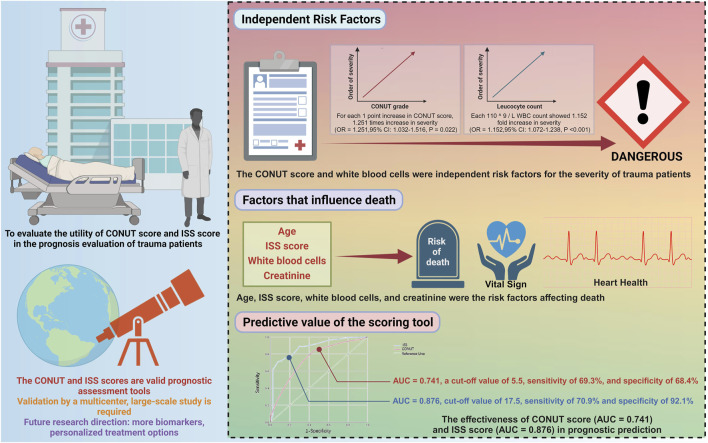
Illustrative application of CONUT and ISS scores in prognosis assessment of trauma patients.

This study discovered that the CONUT score and white blood cell count are independent risk factors for the severity of trauma in patients (Figure 7). This finding aligns with previous research, which has indicated that white blood cell count plays a crucial role in the inflammatory response following trauma, and its levels are closely associated with prognosis. The CONUT score, which assesses a patient’s nutritional and immune status through serum albumin, absolute peripheral lymphocyte count, and total cholesterol concentration, offers unique advantages in evaluating the severity of trauma. These results highlight that nutritional status and immune response significantly impact the prognosis of trauma patients.

In this study, age, ISS score, white blood cells, and creatinine were identified as risk factors influencing mortality in trauma patients. Previous research has also indicated that age is a significant determinant of prognosis for trauma patients, with older patients typically having worse outcomes due to diminished bodily functions and more comorbidities. The ISS score, used to assess the severity of trauma, is widely recognized for its effectiveness in predicting the risk of death in trauma patients. The counts of white blood cells and creatinine levels reflect the state of inflammation and kidney function, respectively. Changes in these physiological indicators are closely associated with the risk of death in patients.

The CONUT score, a simple and efficient nutritional assessment tool, has been proven in this study to effectively predict the prognosis of trauma patients. Compared to its application in other diseases, such as cardiovascular diseases and malignancies, research on the CONUT score in trauma patients is relatively scarce. The findings of this study demonstrate that the CONUT score not only reflects the nutritional status of patients but also serves as an effective tool for trauma prognostic assessment. This discovery provides clinicians with new references for nutritional support and risk assessment in trauma patients.

The ISS is a trauma severity scoring system widely used in clinical practice. This study’s results further validate the ISS score’s effectiveness in trauma prognostic assessment. Consistent with previous research, the ISS score accurately predicts patient outcomes by assessing the anatomical injury locations and the extent of damage. Combined with the findings of this study, the ISS score, as a standardized assessment tool, has once again been confirmed to be crucial in managing trauma patients.

Scientifically, this study enhances our understanding of the relationship between physiological indicators and prognosis in trauma patients. We identified biomarkers closely related to patient outcomes through systematic statistical analysis, such as white blood cell counts and hemoglobin levels. These findings may have important implications for future clinical practice and research directions. Additionally, the use of multiple regression and ROC curve analysis methods in this study also provides an effective analytical framework that can be applied in other types of medical research.

The greatest innovation of this study lies in the first systematic validation of the utility of the CONUT score in prognostic evaluation for trauma patients, addressing a gap where previous research has primarily focused on cardiovascular diseases and malignancies. As a simple and efficient tool for assessing nutritional and immune status, applying the CONUT score in trauma patients has not yet received widespread attention. This study not only confirms its effectiveness in evaluating trauma severity and prognosis but also lays a foundation for its future application across multiple fields. This finding offers a new perspective for guiding nutritional support and immune regulation in trauma patients, especially in high-pressure settings such as emergency departments and ICUs, where simplicity and reliability are particularly crucial. Another significant contribution of this study is the combined analysis of the CONUT and ISS scores, highlighting their complementarity in prognosis evaluation and expanding the application scenarios of traditional scoring systems. In the future, promoting these scoring systems across different populations and trauma types will help further optimize clinical decision-making, ultimately improving patient survival rates and quality of life.

However, the limitations of this study are also quite evident. Firstly, although the sample size is moderate, it is of a single type and mostly concentrated on a specific type of trauma patients, which may limit the generalizability of the results. Additionally, being a single-center study, regional biases may present, and physiological differences in different regions and populations may affect the universality and accuracy of the scoring systems. Furthermore, not all potential confounding factors were explored in the study, which could affect the accuracy and interpretation of the results.

Validating and optimizing the application of the CONUT score and ISS score through multicenter, large-scale studies is essential to enhance their applicability and accuracy among trauma patients of different types and across various regions globally. Additionally, exploring more potential biomarkers that may impact the prognosis of trauma patients and understanding how these biomarkers interact with known scoring systems will be crucial directions for future research. Through these studies, we can gain a better understanding of the physiological changes after trauma and ultimately assist clinical doctors in providing more personalized and precise treatment plans for trauma patients.

## Conclusion

This study comprehensively analyzed 354 trauma patients and confirmed the practicality of the CONUT and ISS scores in predictive assessment. The results show that these scoring tools can be independent factors for predicting adverse outcomes in trauma patients and help clinicians assess patient survival prognoses, thereby guiding clinical treatment. Specifically, applying the CONUT score and ISS score provides clinicians with critical information about patient health status and treatment response, particularly valuable in emergency medical situations.

## Data Availability

The original contributions presented in the study are included in the article/Supplementary Material, further inquiries can be directed to the corresponding authors.

## Abbreviations

AIS, Abbreviated Injury Scale; ALT, Alanine Aminotransferase; AST, Aspartate Aminotransferase; AUC, Area Under the Curve; CI, Confidence Interval; CONUT, Controlling Nutritional Status; CRP, C-Reactive Protein; HDL, High-Density Lipoprotein; ISS, Injury Severity Score; LDL, Low-Density Lipoprotein; MAP, Mean Arterial Pressure; Mean ± SD, Mean ± Standard Deviation; Mon%, Monocyte; NEUT%, Neutrophil Granulocyte; OR, Odds Ratio; ROC, Receiver Operating Characteristic; TBil, Total Bilirubin.
