# Supplementary material for: Association between nutritional status, injury severity, and physiological responses in trauma patients
Source: Front Physiol. 2024 Nov 13;15:1486160. doi: 10.3389/fphys.2024.1486160 (PMC11599220; doi:10.3389/fphys.2024.1486160)
Supplement: Supplementary file 1 [file Table1.docx]

**Table S1. CONUT Scoring Criteria and Assessment Method.**

| **Parameter** | **Scoring criteria** | | | |
| --- | --- | --- | --- | --- |
| Albumin (g/L) | ≥35.0 | 30.0-34.9 | 25.0-29.9 | <25.0 |
| Score | 0 | 2 | 4 | 6 |
| Cholesterol (mg/dl) | >180 | 140-180 | 100-139 | <100 |
| Score | 0 | 1 | 2 | 3 |
| Absolute lymphocyte count (×10^9^/L) | ≥1.6 | 1.20-1.59 | 0.80-1.19 | <0.80 |
| Score | 0 | 1 | 2 | 3 |
| Controlled Nutritional Status Assessment | 0-1(Normal) | 2-4(Mild) | 5-8(Moderate) | 9-12(Severe) |

Note: The CONUT score is calculated by assigning points based on serum albumin, total cholesterol, and absolute lymphocyte count, with the total score obtained by summing these points. The total score reflects the patient’s nutritional and immune status and is divided into four levels: 0-1 (Normal), 2-4 (Mild malnutrition), 5-8 (Moderate malnutrition), and 9-12 (Severe malnutrition).
