# Supplementary material for: Association between nutritional status, injury severity, and physiological responses in trauma patients
Source: Front Physiol. 2024 Nov 13;15:1486160. doi: 10.3389/fphys.2024.1486160 (PMC11599220; doi:10.3389/fphys.2024.1486160)
Supplement: Supplementary file 2 [file Table2.docx]

**Table S2. ISS Scoring Criteria and Assessment Method .**

| **Injured Region** | **AIS criteria** | | | | | |
| --- | --- | --- | --- | --- | --- | --- |
|  | Minor injury | Moderate injury | Serious injury | Severe injury | Critical injury | Untreatable injury |
| Head and Neck | 1 | 2 | 3 | 4 | 5 | 6 |
| Face | 1 | 2 | 3 | 4 | 5 | 6 |
| Chest | 1 | 2 | 3 | 4 | 5 | 6 |
| Abdomen | 1 | 2 | 3 | 4 | 5 | 6 |
| Limbs | 1 | 2 | 3 | 4 | 5 | 6 |
| External | 1 | 2 | 3 | 4 | 5 | 6 |
| **ISS Total Score** | **Assessment Criteria** | | | | | |
| >16 and ≤25 | Moderately severe injury, mortality risk <10% | | | | | |
| >25 and <50 | Severe injury, some mortality risk | | | | | |
| ≥50 and ≤75 | Critical injury, very low survival probability | | | | | |

Note: AIS: Abbreviated Injury Scale. ISS Total Score Calculation: Select the three most severely injured regions of the body. Square the AIS scores for these regions and sum them to get the total ISS score. The total ISS score ranges from 1 to 75.
