# Supplementary material for: Association between nutritional status, injury severity, and physiological responses in trauma patients
Source: Front Physiol. 2024 Nov 13;15:1486160. doi: 10.3389/fphys.2024.1486160 (PMC11599220; doi:10.3389/fphys.2024.1486160)
Supplement: Supplementary file 3 [file Table3.docx]

**Table S3. Distribution of Single Injuries in Trauma Patients.**

| **Injury site classification** | **Number of cases (cases)** | **Proportion** |
| --- | --- | --- |
| Head and neck injuries | 37 | 18% |
| Facial injuries | 4 | 2% |
| Chest injuries | 39 | 19% |
| Abdominal injuries | 28 | 13% |
| Extremity injuries | 100 | 48% |
