# Supplementary material for: Association between nutritional status, injury severity, and physiological responses in trauma patients
Source: Front Physiol. 2024 Nov 13;15:1486160. doi: 10.3389/fphys.2024.1486160 (PMC11599220; doi:10.3389/fphys.2024.1486160)
Supplement: Supplementary file 4 [file Table4.docx]

**Table S4. Baseline Clinical Characteristics of Trauma Patients Evaluating the Effectiveness of CONUT and ISS Scores.**

| **Name** | **Number of cases** | **Proportion** |
| --- | --- | --- |
| Age (years) | 50.7±17.3 | / |
| Male | 268 | 75.7% |
| Female | 86 | 24.3% |
| Hypertension | 47 | 13.3% |
| Diabetes | 25 | 7.1% |
| Coronary heart disease | 7 | 2.0% |
| Cerebrovascular disease | 11 | 3.1% |
| Chronic hepatitis | 7 | 2.0% |
| Length of hospital stay (days) | 13 | / |

Note: The length of hospital stay is calculated as the median for the 354 patients included in this study.
